# CD66b^+^ Tumor-Infiltrating Neutrophil-like Monocytes as Potential Biomarkers for Clinical Decision-Making in Thyroid Cancer

**DOI:** 10.3390/medicina61071256

**Published:** 2025-07-10

**Authors:** Hamdullah Yanik, Ilgin Demir, Ertugrul Celik, Ece Tavukcuoglu, Ibrahim Burak Bahcecioglu, Adile Begum Bahcecioglu, Mehmet Mert Hidiroglu, Sumeyra Guler, Nese Ersoz Gulcelik, Mehmet Ali Gulcelik, Kerim Bora Yilmaz, Gunes Esendagli

**Affiliations:** 1 Department of Basic Oncology, Cancer Institute, Hacettepe University, Ankara 06230, Turkey; yanikhamdullah@gmail.com (H.Y.); ecetavukcuoglu@gmail.com (E.T.);; 2 Department of General Surgery, University of Health Sciences Gulhane Training and Research Hospital, Ankara 06010, Turkey; 3 Department of Pathology, University of Health Sciences Gulhane Training and Research Hospital, Ankara 06010, Turkey; 4 Department of Surgical Oncology, University of Health Sciences Gulhane Training and Research Hospital, Ankara 06010, Turkey; 5 Department of Endocrinology and Metabolism, Gulhane Research and Training Hospital, University of Health Sciences, Ankara 06010, Turkey; begumbahceci@hotmail.com (A.B.B.);; 6 Department of Medical and Surgical Research, Institute of Health Sciences, Hacettepe University, Ankara 06230, Turkey

**Keywords:** thyroid cancer, CD66+ monocytes, cancer diagnosis, monocyte subtypes, neutrophil

## Abstract

*Background and Objectives*: Thyroid nodules are a common endocrine disorder, with 10–15% exhibiting malignancy. Accurate differentiation of malignant and benign nodules is crucial for optimizing treatment outcomes. Current diagnostic tools, such as the Bethesda classification and fine-needle aspiration biopsy (FNAB), are limited in sensitivity and specificity, particularly in indeterminate cases. Tumor-infiltrating immune cells (TIICs) in the tumor microenvironment (TME) play a significant role in thyroid cancer progression. CD66b^+^ neutrophil-like monocytes constitute a novel subset of myeloid cells that are implicated in the modulation of anti-tumor immune responses, but their role in thyroid cancer remains unclear. *Materials and Methods:* Peripheral blood and thyroid nodule tissue samples were obtained from 24 patients with papillary thyroid carcinoma, and from 10 patients who underwent surgery for symptoms of tracheal compression due to benign thyroid nodules. Myeloid cell populations were assayed by flow cytometric immunophenotyping with CD45, HLA-DR, CD14, and CD66b. The data were statistically analyzed with the clinical properties of the patients. *Results:* The neutrophil-like monocytes, which were determined as HLA-DR^+^CD14^+^CD66b^+^ cells, found in the circulation (11.9 ± 2.4% of total mononuclear immune cells) of the patients with papillary thyroid carcinoma, were significantly elevated (*p* < 0.001). Accordingly, these cells were more frequently detected in tumor tissues (21.1 ± 2.1% of total tumor-infiltrating immune cells) compared to non-tumor thyroid tissues (*p* = 0.0231). The infiltration levels of neutrophil-like monocytes were significantly higher in malignant nodules as well as in the peripheral blood of the papillary thyroid carcinoma patients compared to the samples obtained from the patients with benign nodules. The tumor tissues exhibited increased immune cell infiltration and harbored CD66b-expressing neutrophil-like HLA-DR^+^CD14^+^ monocytic cells, which indicates an inflammatory milieu in malignant thyroid cancer. *Conclusions:* This study identifies neutrophil-like monocytes as a potential biomarker for differentiating malignant and benign thyroid nodules. Elevated levels of this novel subtype of immune cells in malignant tissues suggest their role in tumor progression and their utility in enhancing diagnostic accuracy. Incorporating these findings into clinical practice may refine surgical decision-making and improve outcomes through personalized diagnostic and therapeutic strategies, particularly for radioiodine-refractory thyroid cancer.

## 1. Introduction

The frequency of thyroid nodules and thyroid carcinomas has increased in recent decades, making thyroid nodules one of the most commonly encountered endocrine disorders in clinical practice [1,2]. The diagnostic evaluation of thyroid nodules aims to accurately assess their malignancy potential. While 10–15% of clinically identified nodules are malignant, the majority are benign, indicating a relatively low overall risk of malignancy [3,4]. Fine-needle aspiration biopsy (FNAB) remains the cornerstone for evaluating thyroid nodules with suspected malignancy, providing a reliable method for cytological assessment. The Bethesda classification system, which categorizes nodules based on cytological features, plays a critical role in guiding clinical decisions regarding surveillance or surgical intervention [5,6]. Furthermore, genetic testing and molecular analyses have emerged as valuable tools, particularly in cases with indeterminate or suspicious cytological findings, and have become integral components of clinical practice [7]. Recent advances in thyroid cancer biology are rapidly transforming diagnostic and therapeutic paradigms. Identifying reliable biomarkers for clinical application has become pivotal for refining diagnostic algorithms through both germline and somatic testing [8,9]. However, current biomarker testing strategies face significant limitations and disparities, often resulting in suboptimal utility for disease diagnosis and management. Over the past decade, it has become clear that cancer is not merely a collection of tumor cells but rather a complex ecosystem of stromal cells, immune cells, and a tumor microenvironment, emphasizing the critical need to develop robust biomarkers that reflect this intricate tumor microenvironment (TME) and provide actionable insights into thyroid cancer diagnosis and progression [10,11].

The tumor microenvironment (TME) is a dynamic and complex network of immune cells, stromal cells, and extracellular matrix components that collectively shape tumor behavior, drive disease progression, and influence therapeutic responses [12,13,14]. Among tumor-infiltrating immune cells (TIICs), macrophages—referred to as tumor-associated macrophages (TAMs)—are the most extensively studied. TAMs are categorized into two distinct phenotypes: M1-like TAMs, which exhibit proinflammatory and pathogen-eliminating functions, and M2-like TAMs, which promote tissue repair, angiogenesis, and play a central role in thyroid carcinoma progression [15,16]. Similarly, neutrophils have emerged as significant players in the TME, with recent research highlighting their dual role in either promoting or suppressing tumor growth depending on the TME context [13]. Understanding these nuanced interactions is essential for advancing the development of targeted diagnostic tools and therapeutic strategies. Although knowledge about TAMs and neutrophils within the TME of thyroid cancers has expanded significantly over the past decade, our understanding of monocytes—the precursors to macrophages—remains limited.

Monocytes act as a bridge between the innate and acquired immune systems. These cells are influenced by growth factors and cytokines released from the tumor microenvironment, while at the same time, playing an active role in the construction and reorganization of this microenvironment. Under the influence of the cytokines and growth factors they secrete, they help the tumor to grow by angiogenesis and play an active role in the formation of an anti-tumor response by presenting tumor-specific antigens to T cells. In conditions of increased blood cell production, such as cancer and chronic inflammation, there is also a dynamic transition between subtypes of these cells. In the case of monocytes, these subtypes are divided into groups according to the amount of lipopolysaccharide receptor CD14 and CD16 (FC receptor) surface markers. CD14^++^ CD16^−^ cells are referred to as classical, CD14^++^CD16^+^ cells as intermediate transition, and CD14^+^CD16^++^ cells as non-classical monocytes. In addition to these subsets, CD66b+ neutrophil-like monocytes constitute a distinct subgroup of immune cells that are characterized by phenotypic and functional plasticity within the TME [11]. These cells are implicated in promoting tumor progression through mechanisms such as angiogenesis, immune evasion, and extracellular matrix remodeling [17]. Although the active roles of monocytes in the TME have been studied, knowledge regarding their specific role within the TME of thyroid cancer remains limited. While monocyte recruitment and activated inflammation have been implicated in the progression of thyroid carcinogenesis in a preclinical mouse model [18], the specific contributions of CD66b^+^ tumor-infiltrating neutrophil-like monocytes to thyroid cancer remain insufficiently explored [19].

This study aims to elucidate the presence and functional significance of CD66b^+^ neutrophil-like monocytes in thyroid cancer. By assessing the relationship between this cell population within the TME and key clinical aspects—such as the differential diagnosis of nodules, clinical parameters, and disease outcomes—their potential utility as biomarkers in clinical decision-making processes is evaluated. This investigation seeks to provide novel insights into the immunological mechanisms underlying thyroid cancer progression and inform personalized therapeutic strategies.

## 2. Materials and Methods

### 2.1. Patients and Clinical Data

This prospective observational case–control study includes two groups: malignant and benign. The malignant group comprised 24 patients diagnosed with papillary thyroid carcinoma (PTC) based on histopathological evaluation. Tissue samples were collected from the thyroidectomy materials, including the malignant thyroid nodules (tumor tissue) and the adjacent normal thyroid tissue (non-tumor tissue), alongside peripheral blood (PB) samples obtained from the patients.

To minimize potential confounding factors that could affect immune cell populations, patients with a history of systemic steroid use, as well as those with documented rheumatologic or immunologic diseases, were excluded from the study. In the benign group (*n* = 10), a surgical indication was based on the current clinical guidelines and included thyroid nodules larger than 4 cm and those causing tracheal deviation, as evidenced by chest radiography.

PTC samples and PB were obtained from thyroid cancer patients (*n* = 24, median age 55, min.44–max 88, 18 male–6 female) and benign nodules (*n* = 10, median age 51, min 36–max 75, 6 male–4 female). Tissue samples from benign nodules were collected from the thyroidectomy materials, and PB samples were also obtained from these patients. All experiments were conducted after the approval of the local ethics committees and in accordance with the Declaration of Helsinki. Informed consent was obtained from the participants prior to the commencement of the study (Hacettepe University Ethics Committee Approval: GO 21/12-44).

### 2.2. Isolation of Primary Cells

Peripheral blood samples from patients were collected into EDTA vacutainer tubes (BD Biosciences, USA). A diluted blood sample (1:1 with phosphate-buffered saline) was gently layered over Histopaque-1077 (Sigma Aldrich, USA). Histopaque-1077 is crucial for isolating Peripheral Blood Mononuclear Cells (PBMCs) due to its precise density. This solution, with a density of about 1.077 g/mL, perfectly matches the density of mononuclear cells (lymphocytes and monocytes). When a diluted blood sample is carefully layered over Histopaque-1077 and centrifuged, the blood components separate based on their density. Denser components, like red blood cells (RBCs) and granulocytes, settle at the bottom. In contrast, PBMCs, being less dense, form a distinct “buffy coat” layer at the interface between the plasma and the Histopaque. After that, the buffy coat was collected for flow cytometric analysis. For obtaining a cell suspension from the thyroid tumors or non-tumor thyroid tissues, the surgical specimens were finely chopped and incubated in culture media containing 0.075% collagenase type II and 0.01% DNase I at 37 °C. The cell suspension was passed through a mesh with 40 μm pores. Furthermore, 40 μm mesh filtration was used to eliminate cell aggregates and debris. Because the sizes of leukocytes range from 10 to 30 μm, a 40 μm mesh filtration does not remove a certain leukocyte population.

### 2.3. Flow Cytometry

Immunophenotyping was performed with the monoclonal antibodies anti-human CD45 (HI30), CD66b (G10F5), HLA-DR (L243), and CD14 (M5E2) (BioLegend, USA). The percentage of positive cells was determined according to isotype-matched antibodies. Acquiring fully stained multi-color samples, the compensation matrix was applied in real-time or during post-acquisition analysis. This subtracted the calculated spillover from each channel, revealing the “true” fluorescence signal for each marker. The samples were run on Canto II (BD Biosciences, USA), and data were analyzed by FlowJo software (v.10).

### 2.4. Statistical Analysis

Statistical analysis was performed using GraphPad Prism software (v8.1.2) on at least three independent experiments. The methods used for the statistical analyses were one-way ANOVA with post hoc analyses, and a paired or unpaired two-tailed Student’s *t*-test, where appropriate. A value of *p* ≤ 0.05 was considered statistically significant. Otherwise noted, the data are shown as the mean ± SEM.

## 3. Results

### 3.1. CD66b^+^ Neutrophil-like Monocytes Populate the Malignant Papillary Thyroid Carcinoma Tissue

**Figure 1:**
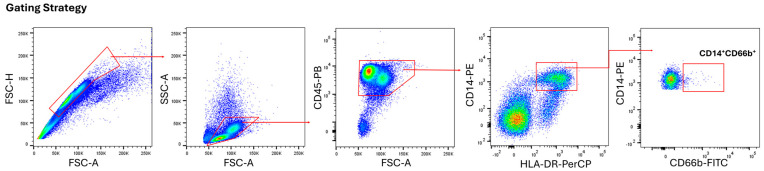
Identification of neutrophil-like monocytes via flow cytometry. FSC-A (Forward Scatter-Area) and SSC-A (Side Scatter-Area), which are the parameters used for the size and granularity of the cells. CD45 is used as a common leucocyte marker. Monocytes were gated as CD14^+^HLA-DR^+^ cells amongst the CD45^+^ total leukocytes. CD14 and HLA-DR positivity were useful for eliminating conventional CD66b^+^ granulocytes and other immune cells, such as T, B, and NK cells. Red boxex and arrows indicate the gated populations.

**Figure 2:**
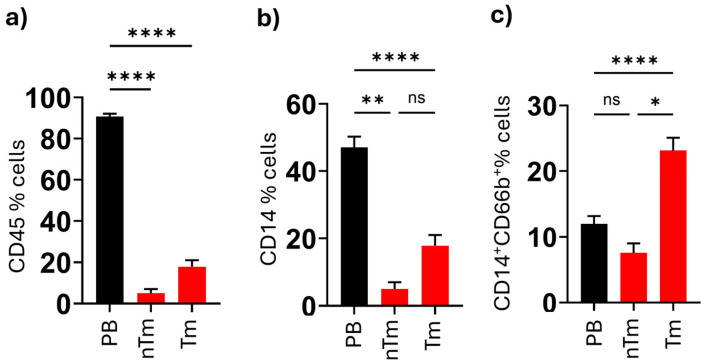
Percentages of total leukocytes (**a**), total monocytes (**b**), and neutrophil-like monocytes (**c**) determined in peripheral blood (PB), non-tumor thyroid tissue (nTm), and malignant tumor tissue (Tm) from thyroid cancer patients. (* *p* < 0.005, ** *p* = 0.004, **** *p* = 0.0001. ns indicates nonsignificant.)

The CD66b^+^ neutrophil-like monocyte population was observed in the non-tumor (tumor-adjacent) thyroid tissue and malignant tumor tissue of 24 PTC patients (Figure 1). The CD45^+^ leucocyte percentage of PB (90.76 ± 2.3%), non-tumor tissue (5.16 ± 2.45%), and tumor tissue (17.86 ± 3.45%) was observed (*p* < 0.001). The HLA-DR ^+^ CD14^+^ monocytes were measured in PB (47.13 ± 3.3%), non-tumor tissue (5.06 ± 1.3%), and tumor tissue (17.08 ± 2.3%) (*p* < 0.001). After that, the HLA-DR^+^CD14^+^CD66b^+^ monocytes were measured in PB (11.9 ± 2.4%), non-tumor tissue (7.5 ± 1.8%), and tumor tissue (21.1 ± 2.1%). The HLA-DR^+^CD14^+^CD66b^+^ monocytes were significantly higher than in the non-tumor tissue (7.5 ± 1.8%) when compared with tumor tissue (21.1 ± 2.1%). (*p* = 0.0231). The HLA-DR^+^CD14^+^CD66b^+^ cells in tumor tissue were 3-fold higher and 10-fold higher when these cells were compared with the PB and non-tumor thyroid tissue. These fold changes could be used as an indicator of the clinical impact of the pathological results. These tumor neutrophil-like monocytes are also significantly higher than the PB neutrophil-like monocytes (*p* < 0.001) (Figure 2).

### 3.2. Comparison of CD66b+Monocyte Frequency Between Malignant and Benign Groups

**Figure 3:**
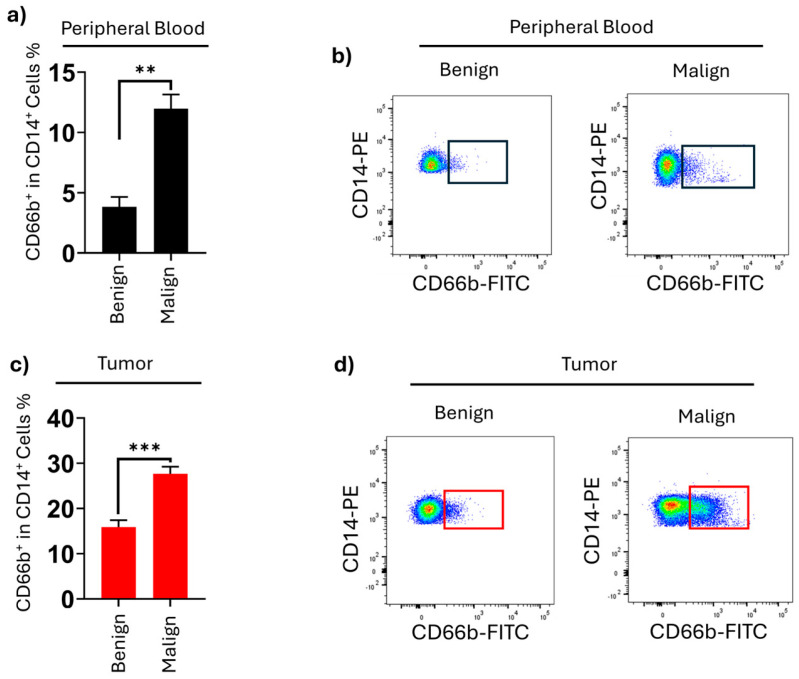
Neutrophil-like-monocyte frequency in thyroid cancer patients with different pathologies in peripheral blood and tumor samples. (**a**) Percentage of CD66b^+^ CD14^+^ monocytes determined in peripheral blood from the patients with benign and malignant thyroid pathologies. Both peripheral blood and tumor. (**b**) Representative flow cytometry results of CD14^+^CD66b^+^ monocytes benign and malignant patients’ peripheral blood. (**c**) Percentile amount of CD14^+^CD66b^+^ monocytes in benign and malignant patients’ tumor tissue. (**d**) Representative flow cytometry results of CD14^+^CD66b^+^ monocytes benign and malignant patients’ peripheral blood. Red and black boxes indicate gated populations. (** *p* = 0.004, *** *p* = 0.0005).

When the HLA-DR^+^CD14^+^CD66b^+^ monocyte percentages were analyzed by flow cytometry using the PB samples obtained from the patients, the HLA-DR^+^CD14^+^CD66b^+^ level of the malignant group (11.9 ± 1.2%) was statistically significantly higher than the benign group (3.8 ± 1.9%) (*p* = 0.0029). On the other hand, the HLA-DR^+^CD14^+^CD66b^+^ monocyte levels, subgroups, and percentages were analyzed by flow cytometry using the thyroid tissue samples obtained from the patients. The HLA-DR^+^CD14^+^CD66b^+^ level of the malignant group (27.6 ± 2.4%) was statistically significantly higher than the benign group (15.8 ± 1.3%) (*p* < 0.001) (Figure 3).

## 4. Discussion

This is the first study to demonstrate the increased levels and functional significance of CD66b^+^ neutrophil-like monocytes in both malignant thyroid tissue and the peripheral blood of papillary thyroid carcinoma patients. Additionally, in PTC patients, we found that the percentages of CD45^+^ lymphocytes and HLA-DR^+^CD14^+^ monocytes were higher in tumor tissue compared to non-tumoral tissue and peripheral blood. The elevated levels of neutrophil-like monocytes detected in malignant nodule tissues and peripheral blood (PB) hold significant clinical relevance for two key reasons. First, these findings underscore the potential of this cell population as a diagnostic biomarker for identifying nodules with a high risk of malignancy, particularly in the context of the Bethesda scoring system, which has known limitations in diagnostic utility. Second, these monocytes may represent a promising immunotherapeutic target, particularly for patients with radioiodine-refractory papillary thyroid carcinoma (PTC), offering new avenues for therapeutic intervention.

Myeloid-derived suppressor cells (MDSCs) represent a heterogeneous population of cells capable of suppressing immune responses, thereby reducing immune activity in cancer [20]. This cell population is typically observed only in conditions of chronic inflammation and cancer and is rarely detected in the blood of healthy individuals. MDSCs are identified by specific surface markers, with monocytic MDSCs (M-MDSCs) and polymorphonuclear MDSCs (PMN-MDSCs) exhibiting low or no HLA-DR expression, which is critical for antigen presentation to T cells. While M-MDSCs are CD66b^−^, PMN-MDSCs are CD66b-positive, and both populations express CD45 [21]. CD66b^+^ neutrophil-like monocytes, as identified in the study by Horzum et al. in TMEs of breast and colon cancers, are a newly identified type of myeloid cell that is not affected by cancer-induced immune-suppressing effects and possesses strong proinflammatory abilities. However, there are currently no established clinical guidelines recommending the routine use of CD66b^+^ monocytes as diagnostic or prognostic biomarkers in any tumor type. Their clinical utility remains investigational and should be validated in larger, prospective studies. Notably, there appears to be no clinical correlation between these cells and specific cancer types or stages, indicating that their presence might represent a common mechanism across different cancers, making them a potential marker independent of the clinical stage [11]. However, whether these cells represent a distinct population or overlap with PMN-MDSCs remains an area of active investigation. While these cells exhibit immunosuppressive properties and contribute to the TME, their clinical relevance appears to be independent of the cancer type or stage, making them a promising marker for further study across various cancers [22].

The comparison between malignant and benign groups highlights the diagnostic value of CD66b^+^ neutrophil-like monocytes. Their significantly higher proportions in both blood and tumor tissue in malignant cases underscore their potential as biomarkers for distinguishing malignant and benign thyroid nodules. Although FNAB and ultrasonography have high diagnostic accuracy rates [23], this biomarker may find a place for use in clinical situations where these methods fail to provide definitive results. Incorporating CD66b^+^ neutrophil-like monocyte levels as a biomarker could enhance diagnostic accuracy and provide critical insights for personalized treatment planning, including surgical decision-making. The alignment of PB results with tumor tissue findings further supports the utility of these monocytes as a supplementary biomarker in diagnostic algorithms. In analyses based on cell counts from peripheral blood, the study found that inflammatory biomarkers such as the neutrophil-to-lymphocyte ratio may provide valuable insights into the role of immune system cells in PB as potential indicators for thyroid cancer [24]. Incorporating biomarkers derived from the TME into diagnostic algorithms, similar to genetic testing [25] and molecular analyses [26], may help overcome the limitations of the Bethesda system. We believe this approach may reduce the risk of missed malignant tumors in false-negative cases and decrease the frequency of unnecessary thyroidectomies.

The differences in CD66b^+^ neutrophil-like monocyte levels between tumor and non-tumor tissues reflect the unique immunoregulatory environment of PTC. Tumors exploit immune cells to create a supportive environment for growth and metastasis [27].The elevated levels of CD66b^+^ monocytes in tumor tissue suggest that these cells are reprogrammed within the TME to adopt a pro-tumor phenotype. CD66b^+^ neutrophil-like monocytes accumulate in the tumor microenvironment, likely in response to tumor-derived signals, including cytokines and chemokines such as IL-8, GM-CSF, and CXCL1. These factors are known to recruit and activate myeloid cells, including monocytes, contributing to the inflammatory milieu and immune cell plasticity observed in various cancers. While these pathways have not been directly investigated in our study, they represent potential mechanisms that warrant further exploration in thyroid cancer. These findings align with previous studies highlighting the plasticity of immune cells, such as neutrophils and monocytes, in response to tumor-derived signals [19,21]. Although CD66b^+^ monocytes exhibit strong proinflammatory properties, their functional role in tumors appears to be context-dependent. In previous studies, such as those conducted in breast and colorectal cancer, these cells have been shown to contribute to tumor progression through mechanisms that include angiogenesis, immune evasion, and matrix remodeling. In our study, we did not conduct functional assays; however, their increased presence in malignant tissue suggests a potential pro-tumorigenic role in papillary thyroid carcinoma.

Investigating components of the TME as potential therapeutic targets is a key focus in cancer research. However, understanding how tumor cells interact with the TME in thyroid cancer remains less explored. In thyroid cancer, the immune microenvironment consists of a range of immune cells, such as T cells, natural killer (NK) cells, mast cells (MCs), tumor-associated macrophages (TAMs), dendritic cells (DCs), and myeloid-derived suppressor cells (MDSCs), as well as secreted immune molecules like chemokines [16]. These components are integral to the onset and advancement of thyroid cancer. The earliest known interactions of the TME in thyroid cancer are related to lymphocytes. In primary thyroid tumors, tumor-associated lymphocytes and a high frequency of regulatory T lymphocytes have been shown to be associated with more aggressive disease [5]. Over the past decade, knowledge has expanded regarding the role of the extracellular matrix and TAM in thyroid cancer. In BRAFV600E-positive PTC, tumor cells recruit stromal cells, including pericytes and fibroblasts, which enhance tumor survival by activating integrins and other prosurvival pathways through secreted factors like thrombospondins and extracellular matrix proteins [20]. Kim et al. showed that in papillary thyroid cancer (PTC) with lymph node metastasis, tumors with higher TAM densities (≥25%) were significantly larger, suggesting a link between TAM density and tumor growth [9]. A study from China showed that TAM density was significantly higher in PTC compared to thyroid goiter and follicular adenoma, with a positive correlation between TAM density and advanced TNM stages (III/IV) [10]. Similarly, the increase in CD66b^+^ neutrophil-like monocyte levels in PTC patients suggests that these cells may have diagnostic value as well as prognostic and therapeutic significance. This immune cell subset could be utilized to classify tumors as “hot” strategies within the TME [23] potentially guiding personalized therapeutic advances in immunotherapy, particularly immune checkpoint inhibitors, which focus on modulating the activity of tumor-infiltrating immune cells. The role of CD66b^+^ neutrophil-like monocytes in shaping the TME suggests their potential as a novel therapeutic target.

Cytokine therapies have been widely studied as a promising approach in cancer treatment due to their ability to modulate the immune system. For instance, cytokines like interferon-alpha (IFN-α) and interleukin-2 (IL-2) have demonstrated efficacy in enhancing anti-tumor immune responses by activating T cells, NK cells, and macrophages [24]. These therapies aim to overcome the immunosuppressive environment of tumors by boosting the activity and proliferation of immune cells [23]. We believe that cytokine therapies aimed at increasing the number of CD66b^+^ neutrophil-like monocytes to enhance anti-cancer immune responses represent a promising and valuable area for further research.

Radioiodine-refractory differentiated thyroid cancer (RAI-R DTC) presents significant challenges in clinical practice. These patients exhibit resistance to RAI therapy, either due to an inadequate iodine uptake by tumor tissue or a lack of therapeutic response [24]. This resistance leads to disease progression, often involving metastases to the lungs, bones, and other distant organs, resulting in increased morbidity and a diminished quality of life. Limited treatment options necessitate the use of systemic therapies for patients unresponsive to surgery and RAI. Multikinase inhibitors (MKI), such as sorafenib and Lenvatinib, have demonstrated potential in suppressing tumor growth and prolonging survival in progressive RAI-R DTC [25]. Additionally, targeted therapies against molecular mutations, such as BRAF and RET inhibitors (e.g., dabrafenib and selpercatinib), have shown efficacy in genetically defined patient groups. Immunotherapies, including PD-1 or PD-L1 inhibitors and MAPK pathway modulators, offer promising options for RAI-R DTC but are yet to become standard in clinical practice [26,27]

One potential limitation of implementing a CD66b^+^ monocyte assessment in clinical practice is the initial cost and technical requirement of flow cytometric analysis. However, compared to advanced molecular or genetic tests, this approach is relatively low-cost and relies on equipment already available in many pathology or immunology laboratories. With standardization and wider adoption, the cost is expected to decrease further, making it a feasible addition to diagnostic workflows, especially in resource-limited settings.

## 5. Conclusions

The emergence of CD66b^+^ monocytes as a distinct myeloid cell subpopulation in the circulation of cancer patients has garnered attention due to their unique molecular and functional characteristics. Unlike myeloid-derived suppressor cells (MDSCs), which often promote tumor progression and immune evasion, CD66b^+^ monocytes exhibit proinflammatory properties that suggest a potential role in enhancing anti-tumor immunity. This study highlights HLA-DR^+^CD14^+^CD66b^+^ monocytes as a significant immune cell population within the TME of thyroid cancer, demonstrating their potential utility as a biomarker for distinguishing malignant from benign cases. These findings enhance our understanding of the immunological mechanisms underlying thyroid cancer and lay the groundwork for the development of targeted diagnostic and therapeutic strategies. Incorporating these insights into clinical practice could significantly improve the accuracy of thyroid cancer diagnoses and lead to better patient outcomes through the implementation of more personalized treatment approaches.

In conclusion, the identification of CD66b^+^ monocytes as a novel myeloid subpopulation with distinct proinflammatory characteristics underscores their importance in the context of cancer. Further research into their mechanisms of action and interactions within the tumor microenvironment could lead to innovative approaches in cancer immunotherapy.

## Data Availability

Data are available upon reasonable request.

## Abbreviations

The following abbreviations are used in this manuscript:

FNAB	Fine needle aspiration biopsy
TIICs	Tumor-infiltrating immune cells
TME	Tumor microenvironment
TAMs	Tumor-associated macrophages
PTC	Papillary thyroid carcinoma
PB	Peripheral blood
MDSCs	Myeloid-derived suppressor cells
M-MDSCs	Monocytic MDSCs
PMN-MDSCs	Polymorphonuclear MDSCs
NK	Natural killer
MCs	Mast cells
DCs	Dendritic cells
RAI-R DTC	Radioiodine-refractory differentiated thyroid cancer
MKI	Multikinase inhibitors
